# Yarn tension control technique for improving polyester soft winding process

**DOI:** 10.1038/s41598-020-79928-1

**Published:** 2021-01-13

**Authors:** Mohamed Ali, Rana Ahmed, Motaz Amer

**Affiliations:** 1 El Sharq El Awsat Co. (STAREGYPT) for Sewing and Embroidery Threads, 2nd Industrial Zone, St. 500 off St. 90, Block 12007, Obour City, 11828 Egypt; 2 Electrical and Control Dept., Arab Academy for Science, Technology and Maritime Transports, Alexandria, Egypt; 3 Basic and Applied Science Dept., Arab Academy for Science, Technology and Maritime Transports, Alexandria, Egypt

**Keywords:** Electrical and electronic engineering, Mechanical engineering

## Abstract

The textile industry has a great role in the improvement of any country’s economy. Moreover, the ready-made garments need different coloured high yarn quality, so yarn should be rewinded on plastic cones for dyeing. However, manufacturers are facing the problem of tension variation during soft winding process that mainly affects the yarn quality. Consequently, to overcome the tension variation drawbacks, the attainment of constant optimal tension values is required in order to: (1) Increase the winding speed while maintaining the yarn quality, (2) Improve the dyeing quality, and (3) Reduce the water consumption during the dyeing process. In this paper, a proposed yarn tension control technique is introduced to upgrade the soft winding machine, thus maintain the yarn quality and improve the manufacturing capacity. The proposed technique has been tested on Polyester yarn samples classified as; fine, medium and coarse yarn counts, to cover most yarn sizes used in the industry. Arduino Mega 2560 controller is utilized to implement the proposed tension control. The results are compared to the conventional system to advocate the effectiveness and capability of the proposed technique in overcoming the trade-off between tension control and machine speed that occurs in conventional system using variable tension levels.

## Introduction

The textile business is known to be one of the oldest businesses worldwide yet, it still needs a lot of efforts and supports. Although it is considered among the poor fields, it acts as an important gate for the countries’ development as it employs a large number of employees with low and medium scales. As known, there are two main fibre families in the textile world namely; natural fibres and man-made fibres. The most famous man-made fibre is the polyester. Most of the garments’ manufacturers choose the man-made fibre in sewing process due to its mechanical performance like; braking force and elongation during high speed sewing processes. Different yarn colours are usually needed for ready-made garments. Hence, in addition to having good quality, yarns shall be also dyed. The Yarn’s quality is related to the thick or thin places and neps resulting from the spinning process. A good yarn should lack those neps. Moreover, the yarn is imported on a paper cone, so in order to make it coloured the paper cone must be replaced by a plastic cone. Here comes the importance of the soft winding machine for both quality improvement and cost reduction^1,2^. Among the unfriendly environmental industry processes, the textile industry processes are the most. During the dyeing process, discharged coloured wastewater massively pollutes the environment. Alternatively, the researchers began to find a solution for the major challenges of having sustainable textile industry with less water consumption and less negative effects on the environment^3–5^.

The yarn dyeing process is undergone in the form of one of two shapes; either the cone shape or the hawk shape. Natural fibres are normally dyed in the hawk shape and under low temperature, so water easily penetrates into the yarn. Oppositely, man-made fibres are dyed in cone shape. Thus, it is more difficult for the water to penetrate through the cone and reach the yarn in the dyeing process. In addition to the global shortage of water, it is challenging for the dyeing manufacturers to reduce the water consumption in the dyeing process for both water and money savings. Subsequently, manufacturers are facing the problem of rewinding the yarn on the plastic cone from the paper cone to begin the dyeing process. Thus, they used to apply a different tension control on the yarn while winding to avoid the existence of any thin places. This, on the other hand, affects the good places as well^6,7^. In this regard, more applicable research is strongly needed in the textile industry to be able to get the best yarn quality, that further improves the textile quality. Different studies were applied on tension control systems in^8–10^. Wang and He^8^ illustrated two tension control technologies, while Eren Recep, İhtiyar Merve, Çelik Özge represented in^9^ the performance of tension control system experimentally using a controlled disc brake. Moreover, in^10^, the imperfection was tested and discussed on the yarn hairiness.

In^11–15^, the researchers developed an image processing-based system to measure the variation in yarn parameters. The performance of the developed system is free from any fluctuation of ambient temperature or humidity. To identify the type and location of yarn periodical errors they used three different signal-processing approaches based on FFT–Fast Fourier Transform, FWHT–FastWalsh–Hadamard Transform, and FDFI–Fast Impulse Frequency. A minicomputer was used for direct digital control, data acquisition, and on-line analysis in addition to the optical sensing run which has been implemented to evaluate yarn quality parameters. Accordingly, more work should be focused on the development of new optimized FDFI algorithms to reduce the associated computational efforts.

Chung et al.^16^ used a splitting machine with an active tension controller to uniformly maintain the tension of the fine yarn. In real experiments, to measure the tension of yarn, a tension sensor is developed using a potentiometer and a tension bar. However, due to the difficulty of using constant PID gains for the tension control, a fuzzy logic based PID controller is proposed. Moreover, fuzzy control strategy is proposed by Liu and Luo^17^ to overcome problems of previous controller and to increase the control accuracy and disturbance resisting ability. Therefore, the fuzzy PID controller for parameter self-adjusting is adopted in this system. In^18,19^, different simulations were applied using fuzzy PID control from different machines manufacturers’ perspectives. Furthermore, reducing the output fluctuation of the controller in accordance with the yarn’s constant tension control system is introduced in^18^. Besides, Minna and Yan^19^ illustrated a fuzzy multiple-attribute group decision making method for the design schemes evaluation of the yarn tension detection and control schemes in rapier looms.

Another different control system has been discussed from the knitting machines’ perspective, where an embedded yarn tension control system is developed by Wu et al.^20^. Zhang et al. explained in^21^, a yarn tension control system based on the design of the sensor manufacturers’ perspective. A new software was designed in which digital motor control and digital display were used. Plus, an easy and simple practical yarn tension controlling system of MCU design was used in its hardware. Moreover, a novel fabric manipulation method is described in^22^ during the sewing process for a fabric control. It addressed the issues encountered in previous attempts concerning tension control and fabric position. In^23–25^, the researchers investigated the relationship between yarn tension and bobbin diameter during the unwinding process. They also developed a software program to read and record the data of the relationship.

Furthermore, the yarn tension and balloon effect were discussed from spinning machine manufacturers’ perspective in^26–28^. The yarn tension is affected by air-drag on the ballooning yarn and the balloon shape leading to yarn breakage, which affects energy consumption and yarn productivity in ring spinning^26^. In^27^, a mathematical model of yarn ballooning motion in ring spinning was established using few methods for real-time measurement of the yarn tension, whereas the dynamical behaviour of the yarn and twist distribution in a modified ring spinning system were investigated by Tang et al.^28^. Moreover^29^, utilized sensors and displayed them to form a comprehensive information management system (IMS) of rapier loom in order to improve the loom’s efficiency and to guarantee improved quality of the textile fabric. The main operating parameters of the loom such as; tension of warp, weft yarns, and weft density, etc. may be adjusted conveniently according to various textile fabrics’ requirements, real time detection, and display of these parameters’ availability. Two new methods of yarn measurement in balloon zone were proposed by Hossain et al.^30^.

Based on an extensive literature review in the textile industry, the tension problem is still an important issue in the soft winding process that hasn’t been completely solved yet. Being a middle process that affects the post processes like; dyeing process, it must grab more research attention. Although researchers designed a yarn tension sensor, it wasn’t used in the soft winding machines in maintaining the yarn quality and solving that difficult problem in the old industry. In addition, most of the research proved that the tension is inconstant. This study proposes a yarn tension control technique to optimize a constant yarn tension over the soft package while speeding up the soft winding machine, which in turn increases the textile manufacturing capacity. The proposed control technique will maintain the soft package winding quality with minimum neps, thins, and thicks, which will help improve the quality of the yarn dyeing process. The improved quality of the soft package will ease the penetration of the dye through the package. Hence, the colour quality of the yarn will be enhanced. In addition, the amount of water used in the dyeing process will be reduced resulting in cost reduction.

The remaining of the paper is organized as follows; section two summarizes the dyeing process and the effect of the winding on dyeing processes. Section three introduces the proposed system technique with equations and flow chart. Section four explains the experimental set-up along with the testing process used to measure the feeder friction equation and the quality indices. Section five then provides a discussion of the results. Finally, section six presents the conclusion of the study.

## Dyeing process

As previously mentioned, the textile industry is one of the most important and rapidly developing industrial sectors that faces two problems; a large amount of water usage and harmful wastewater excretion^31,32^.

**Figure 1:**
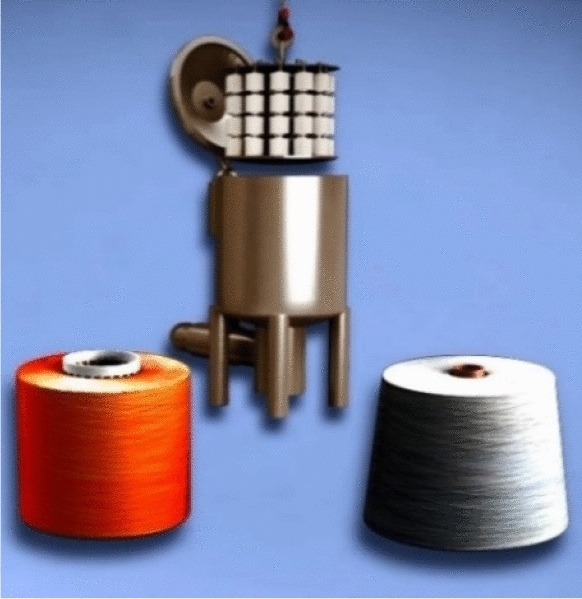
Raw material cone, plastic dyeing tube and dyeing machine [Courtesy of EL Sharq El Awsat Co.].

Moreover, the sewing thread yarn is needed in different colours to meet the needs of the ready-made garments’ manufacturers and craftsmen. The polyester yarn is one of the most important man-made fibres due to its good characteristics in braking force and elongation. But unfortunately, it is imported on paper cone and thus it must be rewind on plastic cone for starting the dyeing process. The dyeing machine used for that process is shown in Fig. 1. Many dyers have different dyeing machines from different generations with different specifications^33^. Also, the dyeing machine is able to work with different liquor-ratios (means the number of litters of water needed for the machine to dye 1 kg of yarn)^34^. Nowadays, there is an increasing attention to the shortage of water as a global environmental problem. On the other hand, textile manufacturers want to maximize their profit through cost reduction by saving the amount of water used for dyeing.

**Figure 2:**
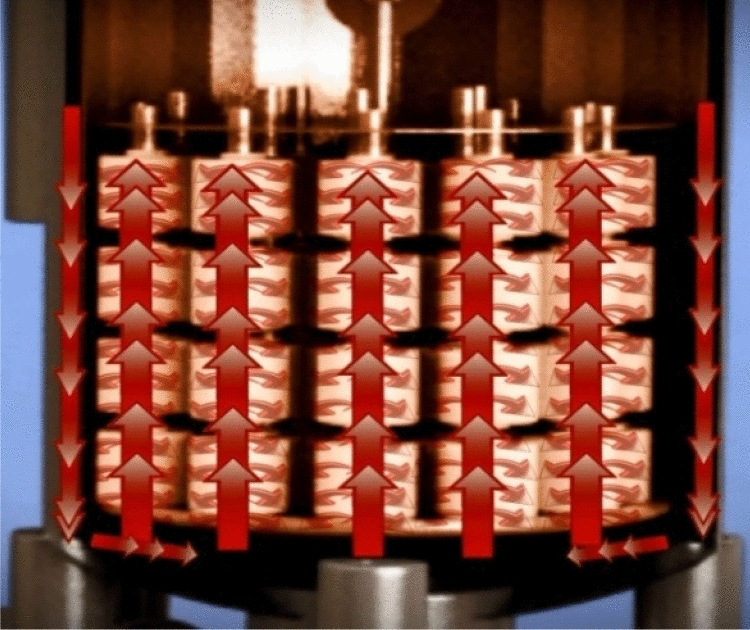
Flow of water from inside the cone to outside [Courtesy of EL Sharq El Awsat Co.].

**Figure 3:**
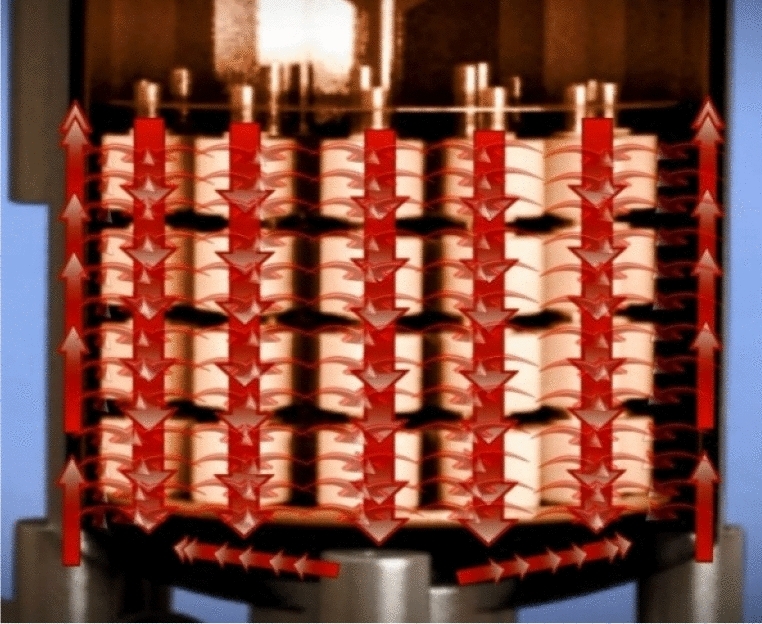
Flow of water from outside the cone to inside [Courtesy of EL Sharq El Awsat Co.].

Normally, the dyeing machine pushes the water using its main pump in two directions; from inside to outside and vice versa, for a certain time. Figures 2 and 3 show the actual direction of water flow in the dyeing machine. However, the latest technique used is to push the water only from inside to outside in order to reduce the amount of water that cover all the carrier during the dyeing process^35,36^.

**Figure 4:**
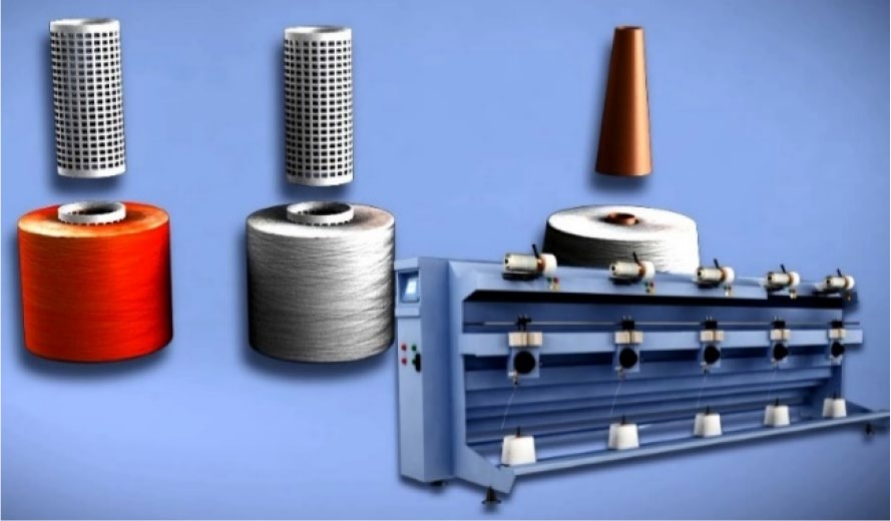
Raw material paper verse plastic cones, and soft winding machine [Courtesy of EL Sharq El Awsat Co.].

Commonly, yarn suppliers provide yarn threads wound on paper cones. Hence, manufactures use soft winding machines to rewind yarn on plastic cones instead for the dyeing process. Therefore, the importance of the soft winding machine (Fig. 4) appears as it is responsible for this transmission. As a result, the non-uniform package diameter problem appears due to using variable yarn tension during the winding process.

**Figure 5:**
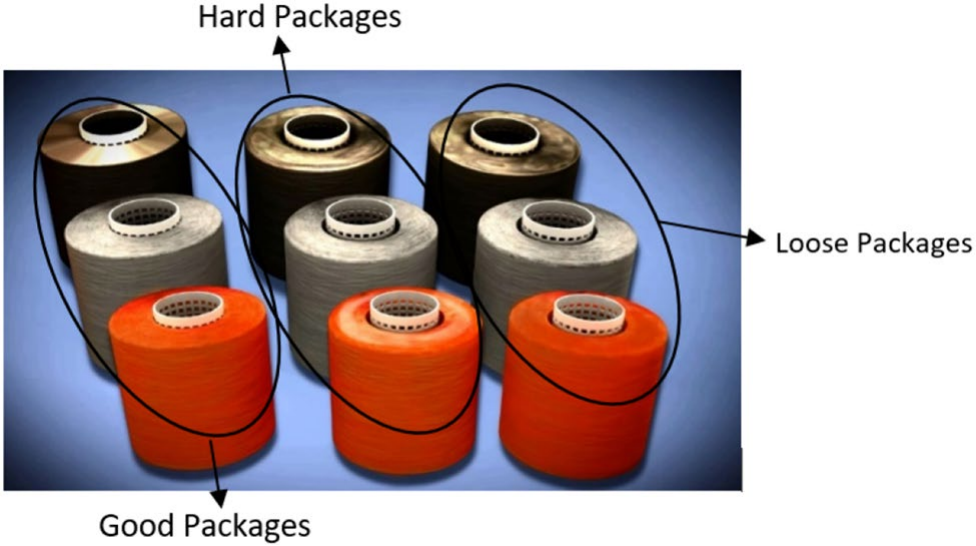
Different densities of soft winding packages [Courtesy of EL Sharq El Awsat Co.].

Consequently, defective products will be produced due to the uneven cone density which further leads to loss of time and money. Figure 5 shows three different density packages and how they get affected by unlevelled dyeing problems that happened due to the soft package deficiency. The first column on the right-hand side shows the loose tension winding problems such as:

1. Off winding problem due to loss of tension in the post process.
2. Affecting the dyeing capacity in the dyeing machine due to the excess dyeing package diameter (the dyeing machine has fixed dimensions), even if it uses a large quantity of water to be dyed.
3. Production of many yarn cuts and reduction in production efficiency.

In the middle column, there are the hard tension packages where:

1. The yarn quality is reduced because hard tension stretches and reduces the yarn size. Thus, yarn quality is affected.
2. The levelling dyeing problem appeared as a reason of hard tension winding that causes difficult dyeing water penetration to the dyeing package (it is much less diameter).

On the left-hand side, the good tensioned packages are shown with:

1. Perfect and optimum compact ready to dye cones diameter.
2. Suitable diameter achieves the optimum dyeing capacity with minimum water usage.
3. The yarn cuts are reduced which results in maintained yarn quality and increased production efficiency.

Furthermore, there is a trade-off between winding tension and winding machine speed. For higher production rate, yarn winding at high speeds may lead to thread break due to increased tension. Therefore, one of the common ways to save the quality is to reduce the winding machine speed, hence reduce the winding tension. Yet, this will lead to productivity reduction. On the other hand, the main problem of the winding process is transferring the yarn from one cone to another while maintaining the yarn quality to enter the post processes with less problems. In turn, it is mandatory to overcome the tension variation that arises due to the different densities of the rewinding plastic and paper cones. Accordingly, the manufactures began to overcome this problem by adding an extra constant high-tension values on the braking disk. The experts noted that the added constant tension is between 1.5–3 cN/Tex, but by adding this extra tension the yarn quality will be affected as it increases its thin places that can be cut easily in the post processes. In this regard, the researchers aim to present an enhanced tension control technique for the yarn winding process. The proposed technique shall provide the following benefits: (1) maintains the yarn quality, (2) improves production, (3) avoids yarn breaking, (4) reduces water consumption and (5) simplify the implementation.

## Proposed yarn tension control technique

In order to study the mechanical and physical model of the soft winding machine, it is preferred to start by tension equations that express each tension used for transferring the yarn from the paper supply package to the plastic cone. First tension is the unwinding tension that can be expressed in Eq. (1)^37,38^.

1 $${\varvec{T}}_{\varvec{unwinding}}={\varvec{m}}_{0}{\varvec{V}}_{\varvec{u}}^{2}\left[\varvec{A}+\left(\varvec{B}\left(\frac{\varvec{z}}{\varvec{c}}\right)^{2}\right)\right]$$

where ${\varvec{m}}_{0}$_,_ is the mass of yarn per unit length (g/cm), ${\varvec{V}}_{\varvec{u}}$, is the speed of withdrawal (cm/s), $\varvec{z}$, is the balloon height (cm), **c**, is the radius at the point of unwinding (cm), **A, B**, are constants depending on climatic conditions, yarn count, inclination of the winding to a plane perpendicular to the bobbin axis, apex angle of the cone, etc.

The weight of the yarn can be expressed by the metric count numbering system which is length in meters per 1 g of weight. Second tension equation comes due to the servo motor “Feeder”, and can be expressed in Eq. (2)^39^

2 $${\varvec{T}}_{\varvec{before \,tension\, brake}} = \varvec{T}_{\varvec{unwinding}} {\varvec{e}}^{-{\varvec{\upmu}}{\varvec{\uptheta}}}$$

where ${\varvec{\upmu}}$, is the coefficient of friction, ${\varvec{\uptheta}}$, is the wrapping angle in radian.

Third tension equation comes because of the electro-magnetic brake. Electro-magnetic tension brake can be expressed in Eq. (3)^40^

3 $${\varvec{T}}_{\varvec{after\, tension\, brake}}={\varvec{T}}_{\varvec{before \,tension\, brake}}+2\varvec{N}{\varvec{\upmu}}$$

where $\varvec{N}$, Number of tension brake, ${\varvec{\upmu}}$, Coefficient of friction.

Fourth tension equation comes from the soft package winding tension (at the upper terminal of the process) as it depends on the diameter of the soft package and its speed. It can be expressed in Eq. (4)

4 $${\varvec{T}}_{\varvec{winding}}=\varvec{M}\frac{{\varvec{V}}_{\varvec{w}}^{2}}{\varvec{R}}$$

where $\varvec{M}$, is the package mass, ${\varvec{V}}_{\varvec{w}}$, is the winding speed, $\varvec{R}$, is the package radius.

The machine is equipped with a damper and a spring to compensate the weight of the final soft package during the winding process, so the mass is neglected. The difference between the input tension and the output tension can be expressed in Eq. (5).

5 $${\varvec{T}}_{\varvec{final}}={\varvec{T}}_{\varvec{before\, tension brake}}+{\varvec{T}}_{\varvec{after\, tension  \,brake}}-{\varvec{T}}_{\varvec{winding}}$$

**Figure 6:**
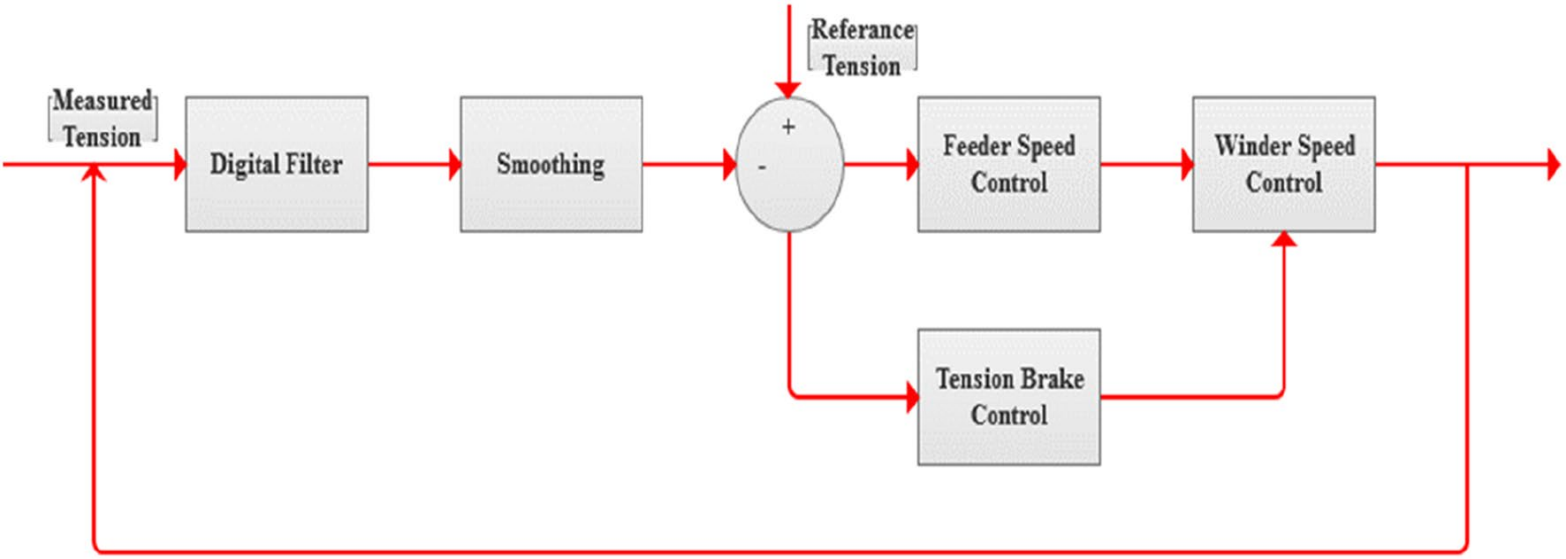
System block diagram.

**Figure 7:**
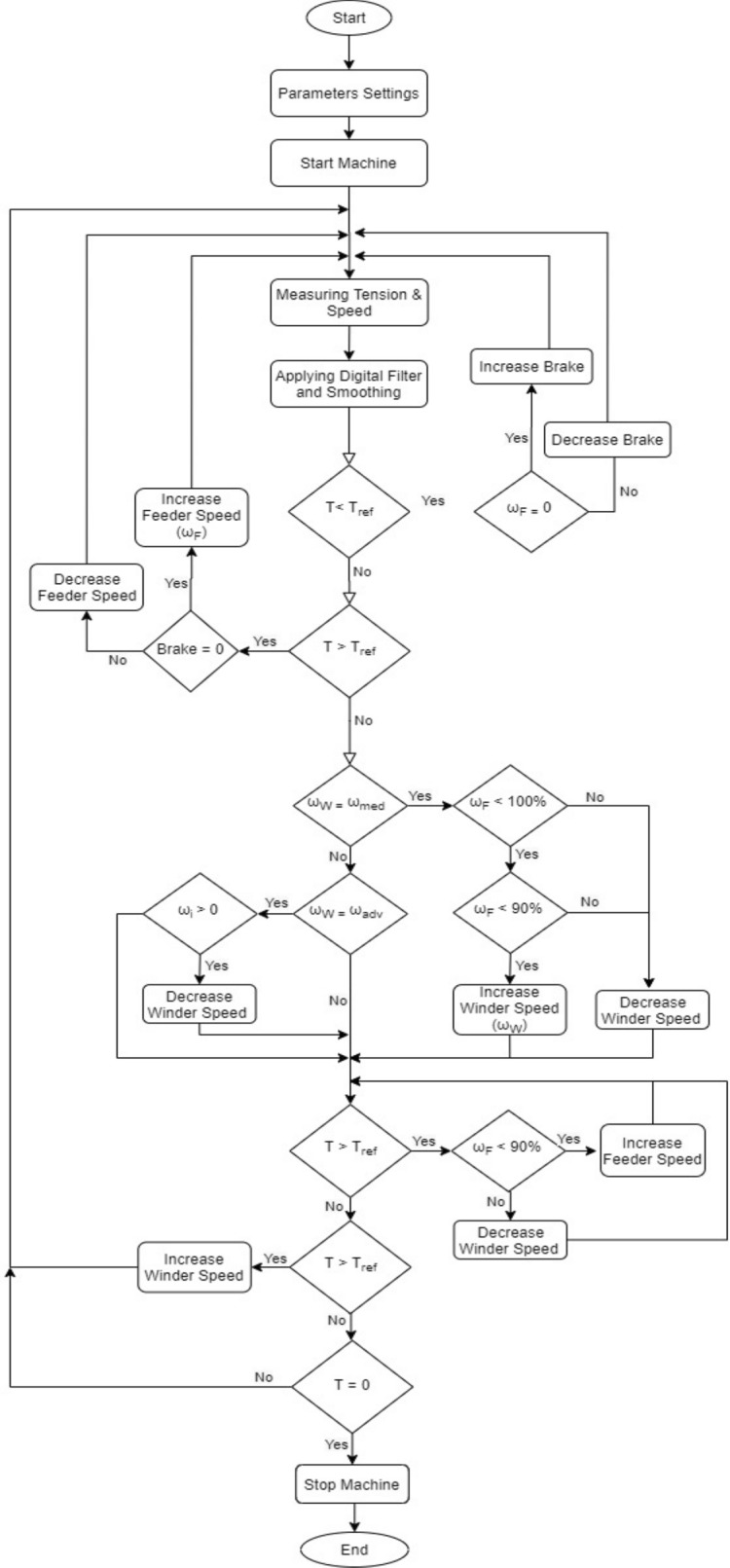
Controller flow chart.

The control system block diagram is shown in Fig. 6 and Arduino Mega controller flow chart is shown in Fig. 7. The flow chart can be split into four sections. The first section shows the parameter setting values of the controller that takes place by using the keypad and LCD. Normally, a memory IC is used to save the parameter setting values but by using the Arduino Mega, it is confirmed that the controller can flash its ROM while executing. Thus, the setting parameter values are protected against the power failure and the controller reset. The controller checks the setting values with the recommended values preprogramed in the software and gives a warning message on the LCD if it is out of ranges. After entering all the setting parameters, the controller waits for the start signal from the winding machine to operate.

The second section represents the measuring step of the controller for the tension using the A/D (analogue to digital port) and the speed in m/min using timer and signals from the proximity sensor. After going through the digital and smoothing step, the controller checks and compares the measured tension with the reference tension.

- Case 1: if the measured tension is less than the reference tension, the controller checks the feeder speed.
  - Case A: if the feeder speed is not equal to 0, then the controller decreases the feeder speed.
  - Case B: if the feeder speed is equal to 0, then increase the electro-magnetic tension brake.
- Case 2: if the measured tension is more than the reference tension, the controller checks the tension of the electro-magnetic brake.
  - Case A: if the electro-magnetic brake is not equal to 0, then the controller decreases tension of the electro-magnetic brake.
  - Case B: if the electro-magnetic brake is equal to 0, then increase the feeder speed.

While, the third section shows the measuring step of the winder speed.

- Case 1: if the winder speed is less than the moderate speed, the controller checks the feeder speed.
  - Case A: if the inverter speed is less than 100% and the feeder speed is less than 90%, then the controller increases the winder speed.
  - Case B: if the inverter speed is not less than 100% or the feeder speed is not less than 90%, then the controller decreases the winder speed.
- Case 2: if the winder speed is more than the moderate speed and less than advanced speed, the controller checks the inverter speed.
  - Case A: if the inverter speed is more than 0%, then the controller decreases the winder speed.
  - Case B: if the inverter speed is equal 0%, the controller stops taking action till it moves to the fourth section.

Finally, the fourth section shows how the controller checks the tension and modifies the speed of the winder with respect to the tension value.

- Case 1: if the measured tension is more than the reference tension, the controller checks the feeder speed.
  - Case A: if the feeder speed is less than 90%, then the controller increases the feeder speed.
  - Case B: if the feeder speed is more than 90%, then the controller decreases the winder speed.
- Case 2: if the measured tension is less than the reference tension, the controller increases the winder speed.
- Case 3: if the measured tension is equal to zero, the controller stops the winder because it means that the yarn is cut.

## Experimental set-up

**Figure 8:**
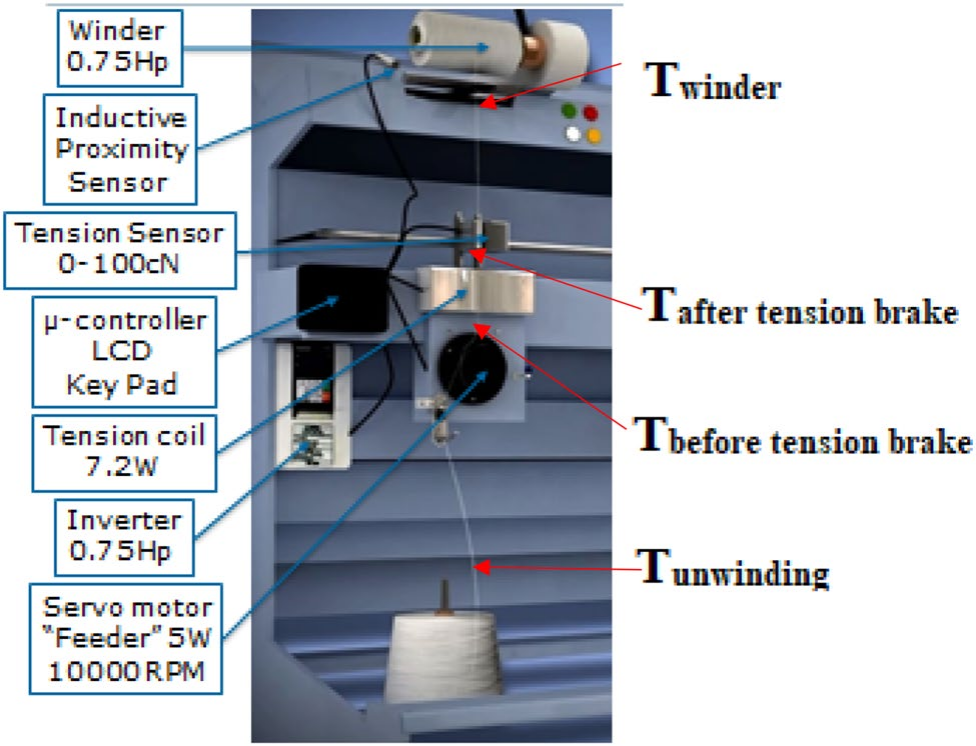
Mechanical model of the soft winding machine [Courtesy of EL Sharq El Awsat Co.].

The new developed yarn tension control system is proposed to solve the tension problems and maintain yarn quality through unwinding process. The tension equations were used during the test samples to define the friction and speed of the servo motor “feeder”. The proposed system has been tested on “Stable Spun Polyester” (SSP) as a man-made fibre example of the textile industrial process. The trials covered different yarn counts including fine count (Ne 50) or (Tex 12), medium count (Ne 40) or (Tex 15) and thick count (Ne 30) or (Tex 20) to cover most of the yarn counts needed in the textile industry. The mechanical model of the soft winding machine that is shown in Fig. 8 consists of:

- The supply package “Raw material”
- The servo motor “Feeder” maximum speed is 10′000 RPM
- The Tension brake using electromagnetic coil
- Tension sensor “piezoelectric sensor”
- Final soft package

**Figure 9:**
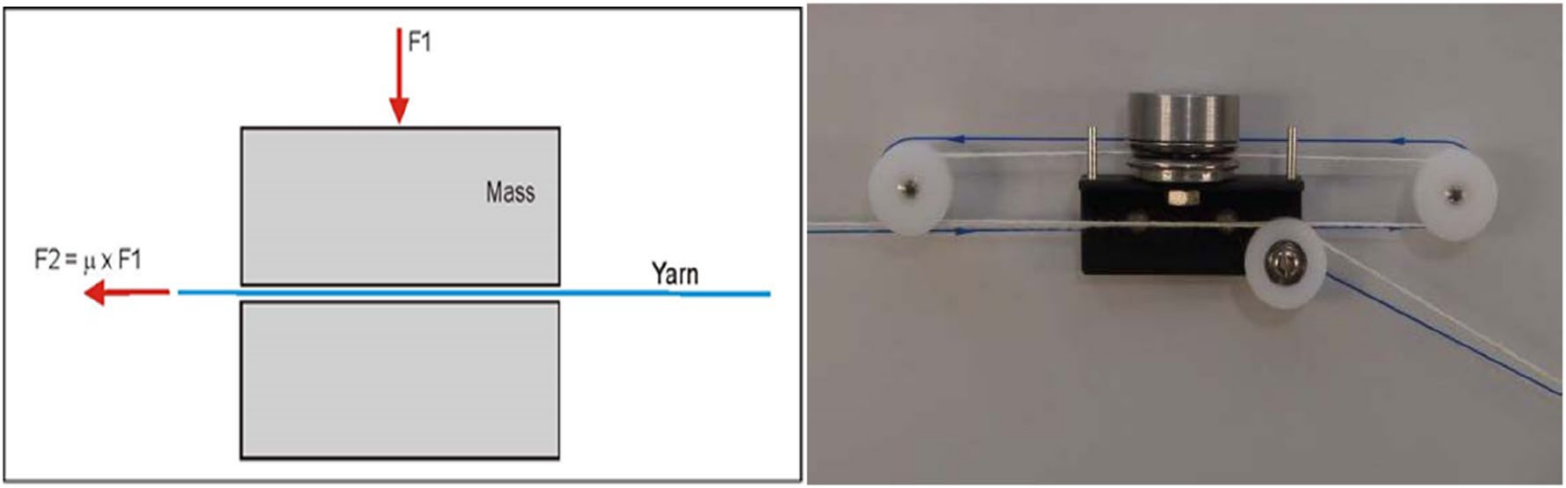
Friction coefficient Tester [Courtesy of EL Sharq El Awsat Co.].

For Eq. (1) the ${\varvec{m}}_{0}$ for each yarn type is as follows, the weight of 1 m of yarn is 0.0118 g for Ne 50. While for Ne 40, weight of 1 m of yarn is 0.0147 g. And for Ne 30, the weight of 1 m of yarn is 0.0196 g. The importance of practical measuring of the yarn friction coefficient used in Eqs. (2) and (3) is illustrated in^41^. Samples have been run over friction tester as shown in Fig. 9. When two surfaces slide over each other, a force resisting motion results; which is called friction. The two basic laws of friction are:

1. The frictional force F2 is directly proportional to the normal load F1 so that the coefficient of friction ${\varvec{\upmu}}$ = F2/F1 is constant for any given two surfaces.
2. The friction is practically independent of the geometric area of contact between the two surfaces.

**Figure 10:**
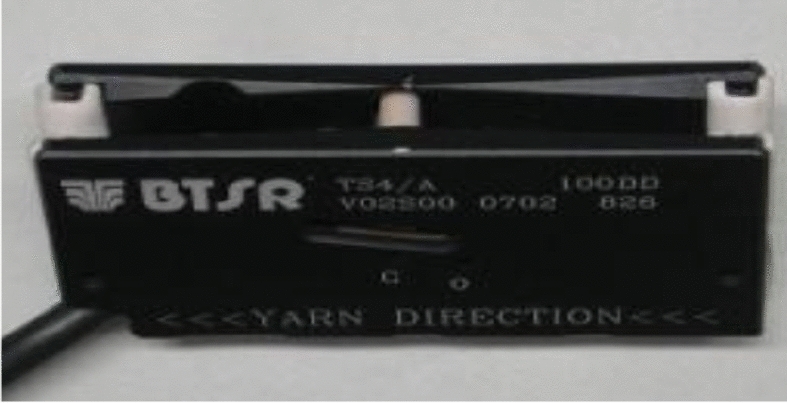
BTSR yarn tension sensor [Courtesy of EL Sharq El Awsat Co.].

Practical measurements of actual tension are illustrated in this section in order to study its behavior. The controller is used to measure the tension values by connecting to the tension sensor through analogue to digital input port (A/D). The results were shown on the LCD attached to the machine. The measuring hardware system has been completed by using BTSR TS44 yarn tension sensor^42^ that is shown in Fig. 10 in stead of using a yarn sensor. It has the advantage of having different wiring connectors and is cheaper than the yarn sensor. Many types of sensors were tested from ELTEX, RETECH and PRIMOSENSOR but proved to be unsuitable for the textile purpose due to the hairiness problem. Hairiness appears when the sensor surface is not smooth and not equipped with ceramic parts in the yarn path. As the yarn is very sensitive, it will act as if it is shaved if it makes a friction with such a hard surface.

### Testing process

Fabric defects and rejections are critically influenced by irregularity of yarn due to the tension used in the soft winding process. Thus, the recommended tension for the yarn plays a very important role and has a great effect on the yarn quality. The evenness of yarn (is a measure of the level of variation in yarn diameter along its length. In other words, it refers to the variation in yarn linear density or mass per unit length of yarn.) is one of the main indices to measure the yarns quality. The unevenness of yarns leads to increased end breakage rate during the spinning and winding process. The increased end breakage rate directly limits the speed of the machines and reduces the productivity. In addition, the unevenness of yarns significantly influences the appearance quality of textiles specially in the sewing process.

**Figure 11:**
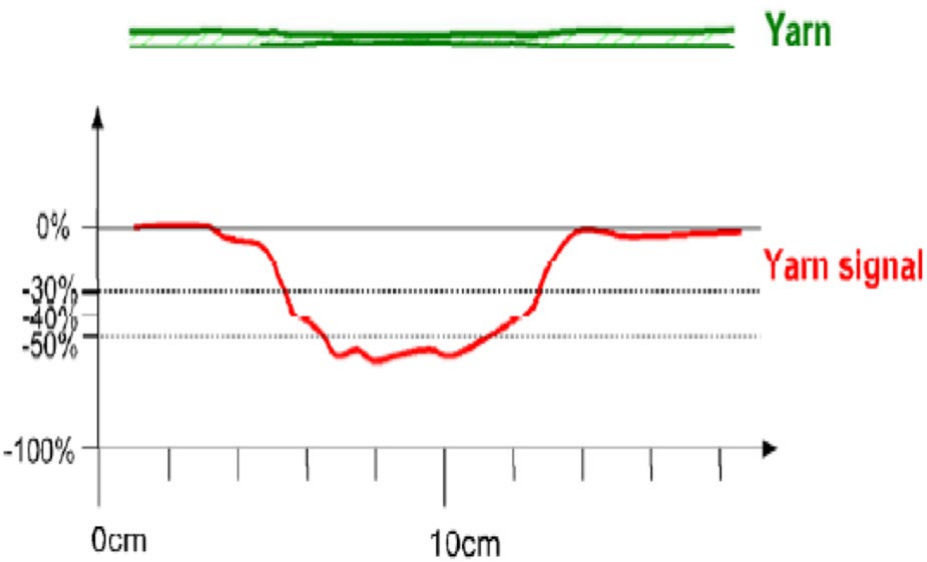
A thin place in a yarn and the corresponding yarn signal.

**Figure 12:**
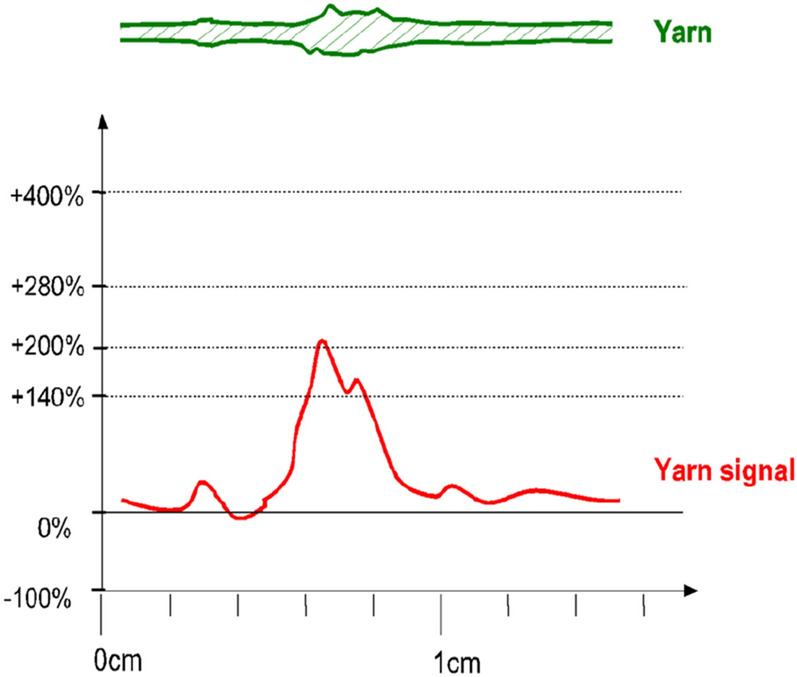
A nep in a yarn and the corresponding yarn signal in the measuring slot.

**Figure 13:**
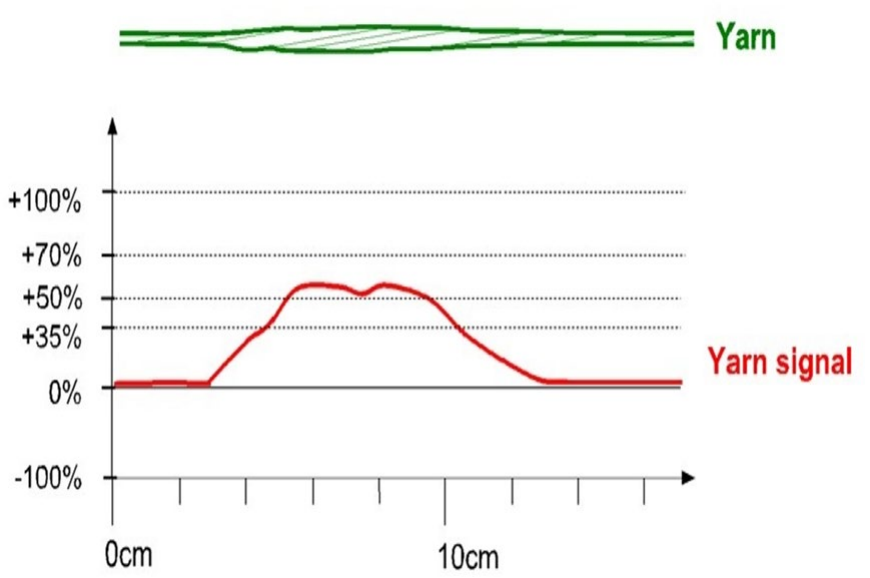
A thick place in a yarn and the corresponding yarn signal.

The three major factors that need to concentrate on the most are thin, thick and neps factors that are expressed in Figs. 11, 12 and 13. The sensitivity thresholds allowed for the thin places are: − 30%/−40%/−50%/−60% so that if the selected limit is exceeded, a thin place is counted. A thin place of −50% means the cross-section area of the yarn in this place is 50% lower than the normal thickness. There is no length limitation for thin places. On the other side, a nep is a very short thick place in the yarn. It can either be made out of fibre material, or trash particles, or foreign matter. The neps sensitivity thresholds allowed are: + 140%/+ 200%/+ 280%/+ 400% where every time the selected percentage value of mass passes the threshold specified a nep is counted. To be noted, the percentage increase for neps is calculated to a reference length of minimum 1 mm and maximum 4 mm. If a short thick imperfection is longer than 4 mm, it is counted either as a thick place or not counted at all; depending on its diameter. A thick place in the yarn usually consists of normal fibre material. Thick place sensitivity thresholds are: + 35%/+ 50%/+ 70%/+ 100%. Thus, thick places of + 50% means its cross-section area is exceeding the normal by 50%. Commonly, the standard sensitive levels are as follows: Thin place: − 50%, Thick place: + 50%, and Neps: + 200%.

**Figure 14:**
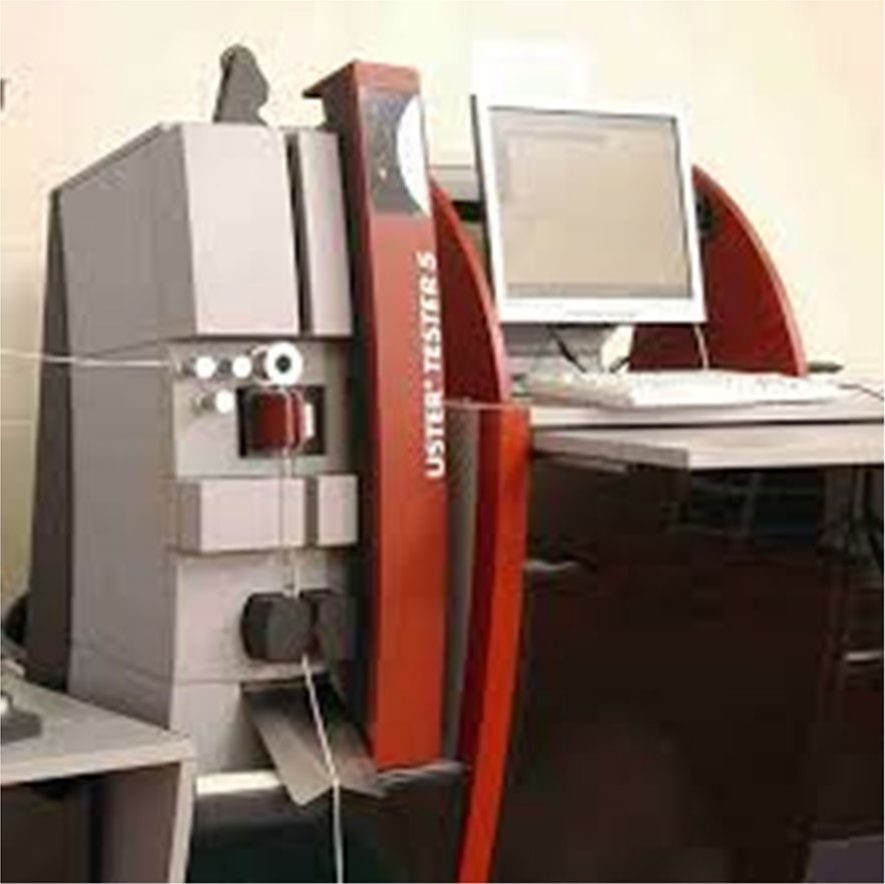
Uster tester five (UT5) [Courtesy of EL Sharq El Awsat Co.].

The Imperfection index (IPI) (which is the summation of all thin and thick places and neps at the standard sensitivity in 1 km of yarn) test has to be applied on the yarn^42^. Samples are tested on a machine called Uster tester five (UT5)^43,44^, as shown in Fig. 14. This machine is responsible for measuring all parameters that affect the yarn quality such as; unevenness of yarn (U %), coefficient of variation of mass (CV_m_ %), roving sliver, and imperfection index. All measurements and calculations have to be done for each recommended tension to assure the yarn quality.

**Figure 15:**
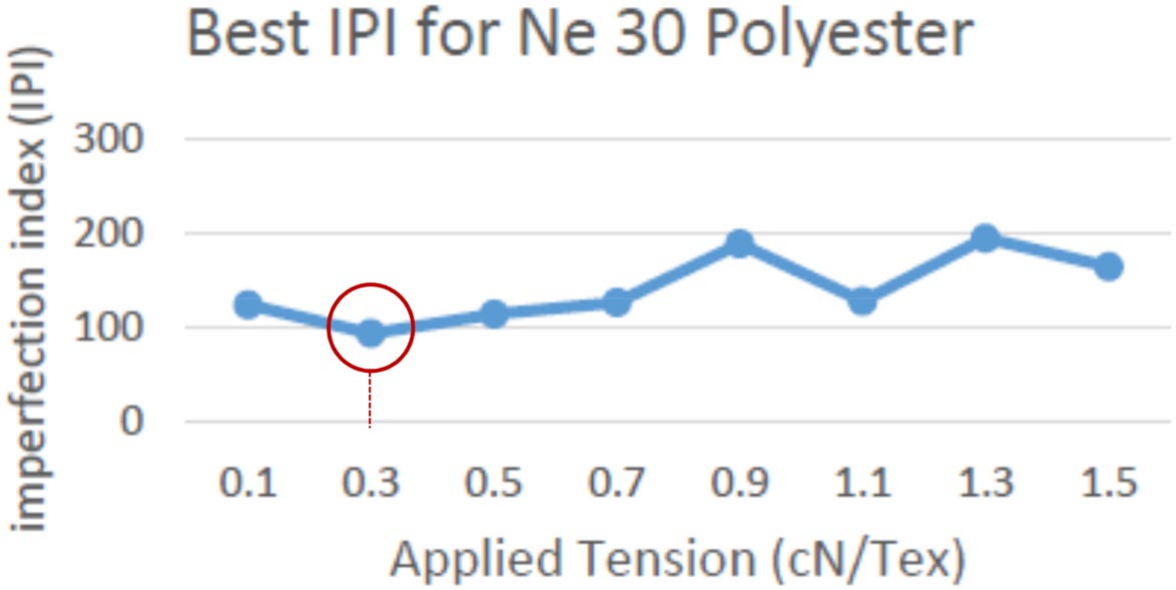
Best IPI for yarn count Ne 30 polyester yarn.

**Figure 16:**
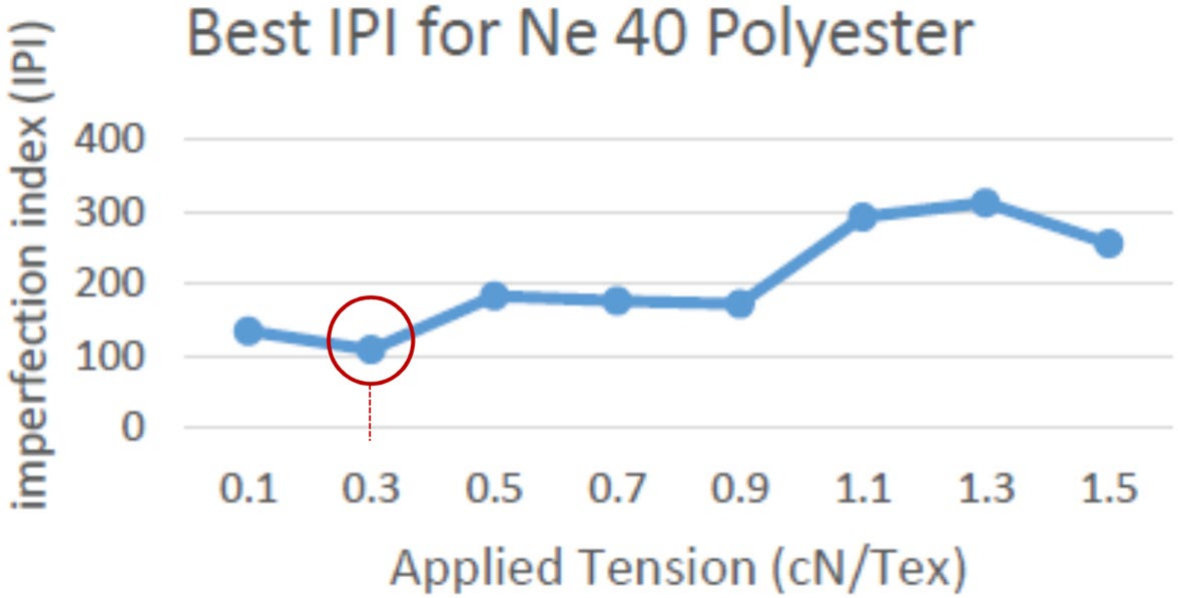
Best IPI for yarn count Ne 40 polyester yarn.

**Figure 17:**
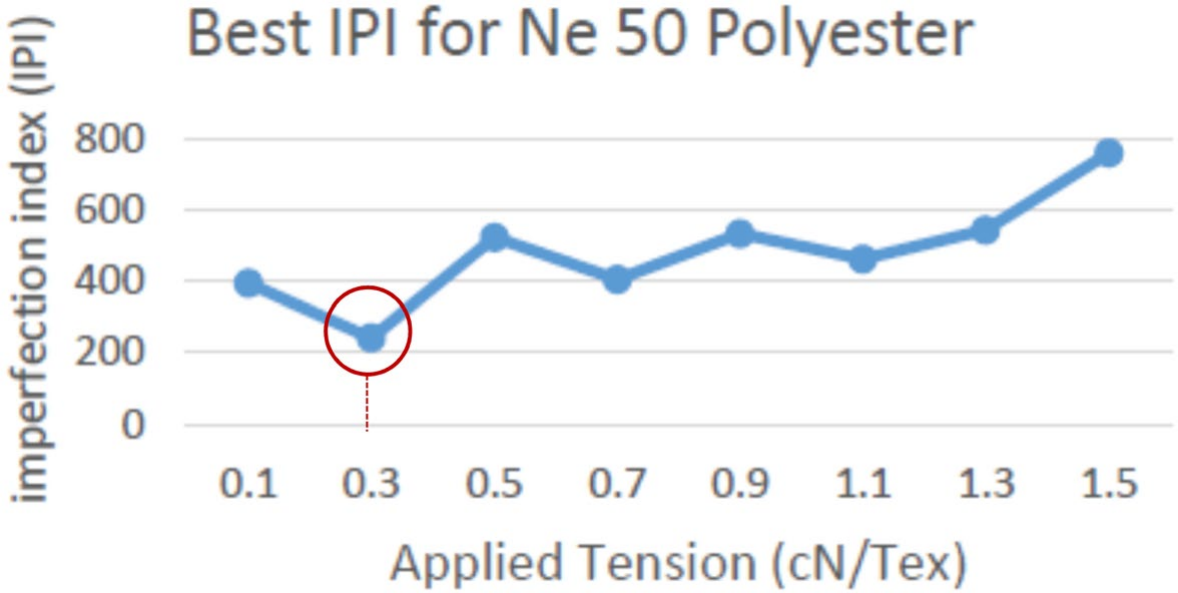
Best IPI for yarn count Ne 50 polyester yarn.

Different practical trials were applied to get the optimum tension value (when IPI is nearest to 100%; which means having the minimum yarn defections) using the proposed technique for maintaining the yarn quality. The Imperfection Index has been tested with different applied reference tensions on the soft winding machine, in addition to increasing the running speed up to 1200 m/min instead of normal winding speed (between 900 m/min and 1000 m/min). Tests were applied on Ne 30, 40, and 50 polyester yarn respectively, and the results are shown in Figs. 15, 16 and 17.

## Results and discussion

**Figure 18:**
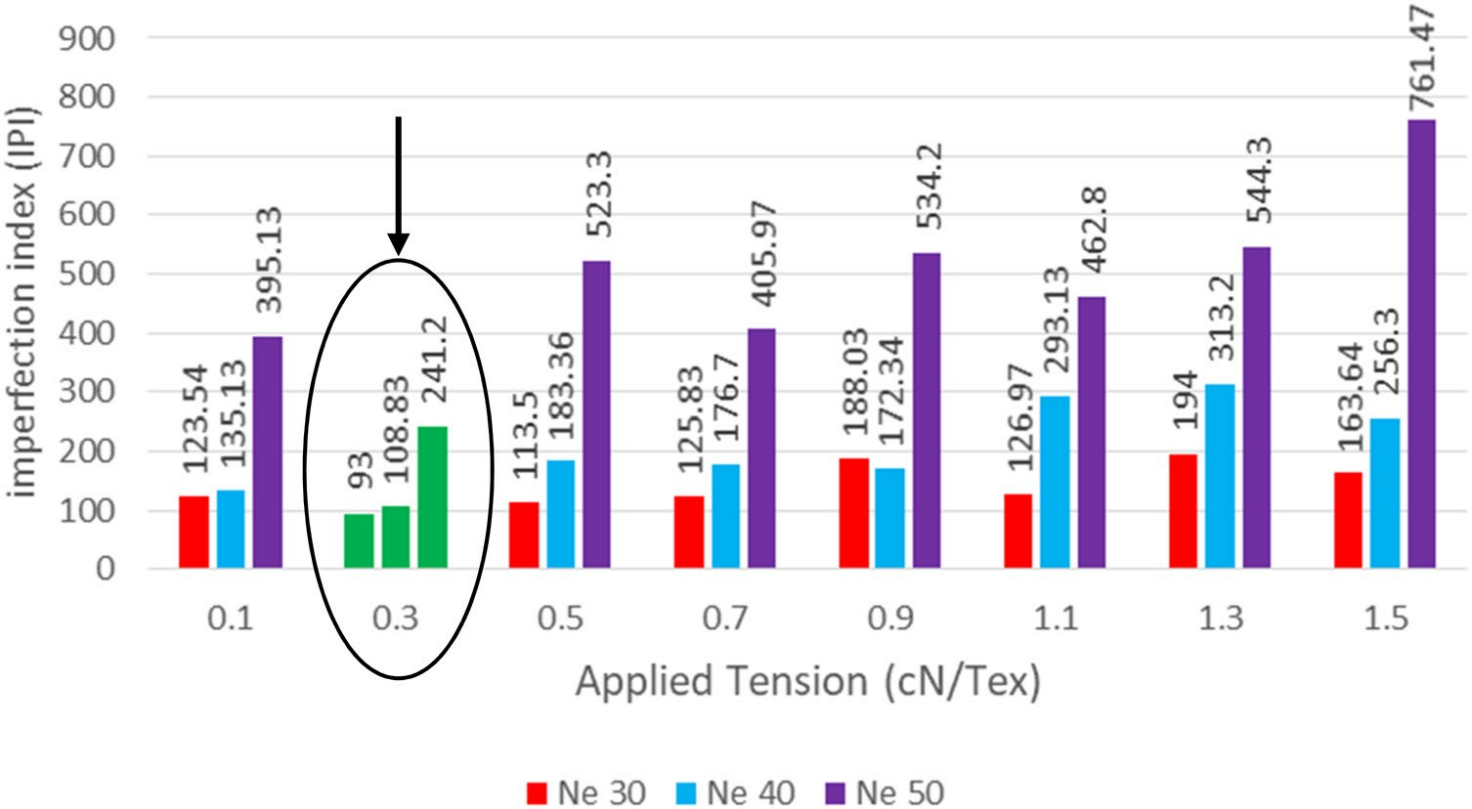
Total Best IPI for yarn count Ne 30, 40, 50 polyester yarn.

The above definitions and tests proved the success of using the new proposed yarn tension control technique in controlling the tension on a real soft winding machine. Figure 18 illustrates the applied tension results for different polyester yarn; where the x-axis represents the different tension values applied on the yarn and the y-axis represents the total number of imperfection index problems (summation of thin and thick places, and neps). The precise and accurate measured results confirmed that the optimum constant tension for the man-made fibre (spun polyester); without additional tension rates between 1.5–3 cN/Tex, is 0.3 cN/Tex as it achieved the minimum number of IPI. Also, it is found that the best imperfection index (IPI) is not related to the yarn count but it is determined by the fibre type.

**Figure 19:**
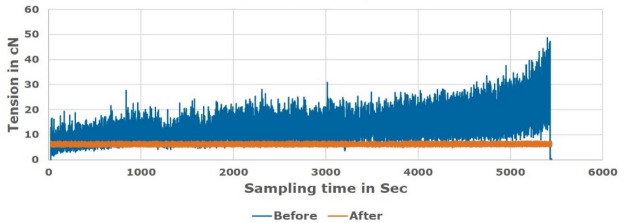
Unwinding tension before and after proposed system for Ne 30 polyester yarn.

**Figure 20:**
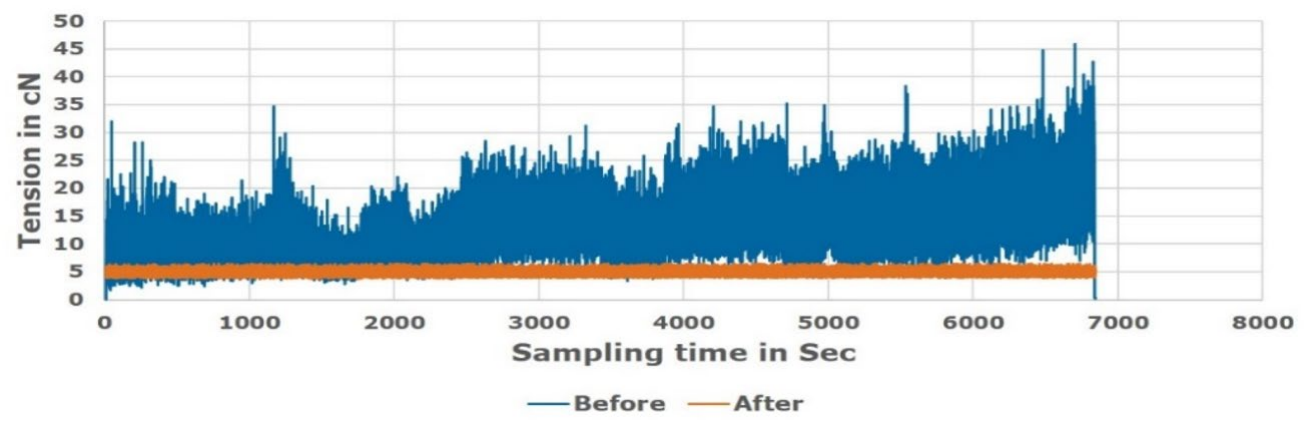
Unwinding tension before and after proposed system for Ne 40 polyester yarn.

**Figure 21:**
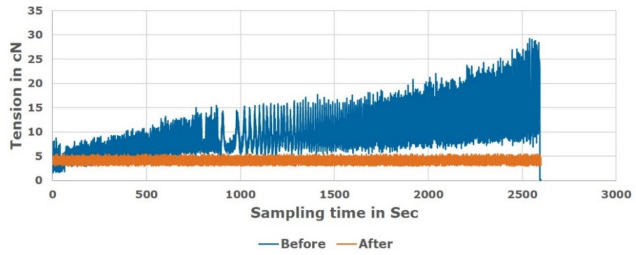
Unwinding tension before and after proposed system for Ne 50 polyester yarn.

Figures 19, 20 and 21 showed a comparison of practical measuring results applied for Ne 30, 40, 50 polyester yarn respectively during the actual manufacturing process. The comparison showed the difference in the tension behaviour between the new proposed constant tension control technique and the conventional system that uses added tension values.

The study was conducted on a moderate (average) supply package diameter. Different tests were applied on the soft winding machine using the polyester samples to know the friction and speed effects of the servo motor “feeder”. The tests speed was varying from 750 m/min up to 1500 m/min. However, due to the machine vibration, the speed is optimized to be 1200 m/min; which means about 25% average increasing above the nominal speed.

The comparison validated that the proposed technique succeeded in saving the optimum tension constant during the winding process in contrast to the variable tension that appears in the conventional winding process. Therefore, it is noted that the new proposed tension control system works effectively and the tension is controlled without any problems. Target tension values are achieved successfully and the quantity of water used during the dyeing process is reduced by about 70%. That is due to the uniformity of the package after transferring from the paper cone to the plastic cone as a result of using the optimum constant tension in the winding process.

The proposed technique allowed the soft winding machine to operate up to 1200 m/min which is for about 20–33.3% higher than the maximum common operating speed range (900 m/min–1000 m/min). It also saved about 70% of water during the dyeing process due to package uniformity (as the 1 kg of yarn normally consume about 10 L of water, but after applying the proposed technique it has been reduced to 3L/kg). Therefore, improved production, reduced water consumption, lower costs, and maintained quality of the yarn, were successfully achieved using the proposed optimum constant tension control technique.

## Conclusion

The proposed yarn tension control technique maintains the yarn quality. The value of 0.3 cN/Tex is the new optimum recommended tension value for polyester that maintains yarn quality with best IPI as advocated by experimental results. The proposed technique results in speeding up the soft winding machines up to 20–33.3% higher than the maximum common operating speed (900–1000 m/min) with respect to the optimum tension control. Besides, it leads to better build up soft package production with shorter time span, enhanced dyeing process quality and increasing manufacturing productivity while reducing the water quantity used in the yarn-dyeing process by 70%. The proposed yarn tension technique can be implemented on both new and existing machines with the ability to know the recommended tension for any new samples. The proposed tension control technique contributes to the overall system cost effectiveness by replacing costly yarn sensor with cheaper market ready tension sensor.
